# Reaching common ground: Protocol for a qualitative study to explore Korean immigrant patient experiences in shared decision-making with primary care physicians

**DOI:** 10.1371/journal.pone.0354905

**Published:** 2026-08-07

**Authors:** Kyla Gaeul Lee, Anna R. Gagliardi, Aisha K. Lofters, Andrew D. Pinto, Robert G. Maunder

**Affiliations:** 1 Institute of Medical Science, Temerty Faculty of Medicine, University of Toronto, Toronto, Canada; 2 Department of Psychiatry, Sinai Health, Toronto, Canada; 3 Toronto General Hospital Research Institute, University Health Network, Toronto, Canada; 4 Department of Family and Community Medicine, University of Toronto, Toronto, Canada; 5 Upstream Lab, MAP Centre for Urban Health Solutions, St. Michael’s Hospital, Toronto, Canada; Universiti Putra Malaysia, MALAYSIA

## Abstract

Shared decision-making is central to patient-centered care, yet immigrant patients, particularly East Asian immigrants, are underrepresented in shared decision-making research. Cultural norms and language barriers may shape the decision-making experiences and preferences of East Asian immigrants. Further, existing research on East Asian immigrant patient preferences and experiences is often non-purposive and conflicting. This hermeneutic phenomenological study explores Korean immigrant patients’ experiences and preferences in their therapeutic relationships with Canadian primary care providers, focusing on the elements of “caring” and “sharing” in shared decision-making. Twelve adult Korean immigrant participants will be recruited through a purposive criterion and convenience sampling approach via both active and passive recruitment methods. Participants must have or have had permanent resident status in Canada, have immigrated to Canada from South Korea in the economic category, and have a family doctor or have seen the same primary care physician at a walk-in clinic at least three times. Interviews will be conducted in Korean or English and analyzed through an inductive analysis approach. This study is currently in the recruitment stage. Findings will be shared to participants, the communities involved and published in a peer-reviewed journal presented as aggregate, de-identified themes.

## Introduction

Shared decision-making (SDM) is a process that promotes patients’ involvement in their own care by respecting and responding to their individual preferences, needs, and values [1,2]. It is endorsed by professional bodies in Canada such as the Royal College of Physicians and Surgeons of Canada, which identifies SDM as a core competency in their Physician Competency Framework. Key competencies for SDM include establishing therapeutic relationships with patients, eliciting relevant information from patients, incorporating their perspectives, sharing healthcare information and plans, and engaging patients and their families in developing plans that reflect the patient’s healthcare needs and goals [3].

Central to SDM is the therapeutic relationship, which creates an environment where patients feel respected, listened to, and valued [4–6]. This relationship can be described as a partnership, or bond, between the provider and patient, composed of two elements: “caring” and “sharing” [7–10]. Research shows that a strong therapeutic relationship based on “caring” and “sharing” can enhance healthcare outcomes, including increased patient and provider satisfaction and improved quality of life [11–13]. “Caring” is characterized by trust, positive rapport building, empathy, and connection [5,14–16], while “sharing” reflects key competencies of SDM [17], where providers and patients jointly set goals and action plans based on the patient’s priorities and agree on respective roles within the relationship [18–22]. This then enables the provider and patient to reach an agreement and shared understanding, or common ground, regarding the patient’s healthcare.

Reports published since the Institute of Medicine’s recommendation in the early 2000s indicate that most patients prefer sharing decisions with their providers [23]. However, these reports may not reflect the perspectives of immigrant patients, who remain underrepresented in the SDM literature [24]. Immigrant patients often have cultural [25,26] and linguistic [25,27,28] differences from their providers, which may shape preferences and experiences distinct from those of native-born patients. For example, there are conflicting reports regarding the preferences of East Asian (Chinese, Japanese, Korean) immigrant patients in participating in SDM and engaging in the therapeutic relationship to establish mutual understanding. While some studies report that East Asian patients prefer to defer decision-making to their providers in their therapeutic relationships [29–34], others report preference for a more active approach to participating in decision-making [35–38]. Many East Asians come from cultures that value hierarchy and respect authority, which may lead patients to prefer a more passive role in the therapeutic relationship and decision-making. When physicians offer options and choices, it may be perceived by East Asian immigrant patients as indicating a lack of expertise and authority [39,40]. Conversely, patients who are more acculturated may prefer to engage more actively in the therapeutic relationship [40]. Even among those favoring a passive role, many still wish to receive sufficient information and establish mutual understanding with their providers [29,40,41]. Misinterpretation by providers can further complicate SDM; assumptions that East Asian immigrants prefer passive roles in decision-making may stem from language barriers, patients’ difficulty articulating preferences, time constraints, or power imbalances in the patient-provider relationship [42,43]. Such miscommunication is not limited to East Asian populations. For example, one study of Arabic immigrant patients found that some believed “involvement” in decisions meant making healthcare decisions entirely on their own without provider input [6,44].

Given the significant role of immigration in shaping Canada’s population, it is crucial to better understand East Asian patients’ experiences and preferences for healthcare decision-making. By 2041, it is estimated that half of the Canadian population will be immigrants or children of immigrants born in Canada [45]. Furthermore, Asian countries account for 7 of the top 10 countries of birth of recent immigrants [46]. Immigrants drive almost 100% of Canada’s labor force growth, with the majority of immigrants from the economic immigration category [47]. Although immigrants typically enter Canada in good health, numerous studies report the healthcare experiences of immigrants to be marked with barriers, leading to a later deterioration of health [25,26,48–51]. As a significant proportion of the Canadian population consists of individuals with immigrant backgrounds, it is essential to explore, and better understand, relationships between immigrant patients and their physicians.

According to the 2016 and 2021 Canadian Census, South Koreans are the second largest East Asian immigrant group after those from China [52,53]. Yet, research on healthcare decision-making among Korean immigrants in Canada, particularly within primary care, remains limited. Although many East Asian cultures have Confucianist roots, they are not culturally monolithic; preferences and experiences in decision-making can vary considerably between cultural and national groups [25,54], meaning findings from one East Asian immigrant population may not be directly applicable to another. The literature on SDM among East Asian immigrants as a whole is sparse, and most existing studies have focused on oncology or end-of-life contexts, which often involve different decision-making dynamics than primary care. This has created an evidence gap regarding East Asian immigrants’ attitudes toward SDM in primary care, with especially little information available for South Korean immigrants. As SDM emphasizes prioritizing patients’ unique and individual preferences and values, the wishes of patients who prefer physician-led decision-making should be respected, provided that they are informed about the various decision-making styles and roles.

Primary care is particularly well suited to investigating SDM and the therapeutic relationship. Serving as the gateway into Canadian healthcare, it encompasses a wide range of services, from treatment and referrals to reassurance and education [55–58]. Because patient-provider relationships in primary care often span years or even decades, primary care allows for the development of strong therapeutic relationships, an essential foundation SDM [56].

Therefore, the overall aim of the current study is to explore the experiences and preferences of Korean immigrant patients in their therapeutic relationship with Canadian primary care providers. Conceptually, the therapeutic relationship is composed of “sharing” and “caring” elements [9,10]. Therefore, to address this aim, the study will pursue two specific objectives: (1) to explore what Korean immigrant patients consider important in the therapeutic relationship, specifically focusing on the elements of “sharing” and “caring”, and (2) to investigate Korean immigrant patients’ experiences and preferences regarding the decision-making process (e.g., identifying the need for a decision, deliberation and weighing options, and reaching a final decision) with their primary care providers.

## Methods

### Study design

The design of the current study is qualitative. Qualitative research explores and provides insights into human behavior and real-world problems by gathering data on participants’ experiences, perceptions, and behaviors [59,60]. Research objectives that seek to understand the “why” and “how” of experiences and perceptions are inherently difficult to quantify, but can be effectively explored using qualitative methods [59]. Therefore, this study will take a qualitative approach to address the research objectives outlined above. More specifically, a hermeneutic (or interpretivist) phenomenological qualitative approach will be taken.

### Theoretical and conceptual underpinnings

#### Qualitative approach.

The current study aims to explore the above objectives through a hermeneutic phenomenological approach. Phenomenology attempts to “make explicit the implicit structure and meaning of human experiences” [61,62] and seeks to understand the participant’s world as they live it [63]. In doing so, the findings of a phenomenological study is a collection of descriptions of meanings (i.e., experiences, preferences) individuals attribute to their lived experiences of a concept or phenomenon [64] (i.e., decision-making in primary care). Hermeneutic phenomenology views people and the world as indissolubly related and tied in cultural, social, and historical contexts [65,66]. Hermeneutic phenomenology considers the positionality of the person in their social, cultural, and historical contexts in interpreting their realities and lived experiences, and their experience of the phenomenon. Given that the aim of the current study is to explore the therapeutic relationship and SDM, which are inherently personal, interpersonal, and situated in social settings, from patients’ perspectives, hermeneutic phenomenology is a well-suited approach for this study. By adopting a phenomenological approach, this study seeks to describe participants’ lived experiences and preferences in decision-making and the therapeutic relationship, which may be influenced by their cultural background and status as an economic immigrants. However, as hermeneutic phenomenology claims that meaning is found as people construct the world and relevant knowledge based on their own background and experiences [65], even those who share similar cultural, ethnic, and immigration backgrounds may have different experiences and preferences relating to decision-making and the therapeutic relationship.

In qualitative research that takes an interpretivist framework of inquiry, validity and reliability are achieved through the rigor, trustworthiness, credibility, and authenticity of both the research and the researcher [65]. Therefore, reporting and deeply reflecting on the researcher’s experiences and assumptions are critical for the research process. The first author and research lead of the study will report their positionality. Hermeneutic phenomenology asserts that the observer or researcher cannot fully remove themselves from the phenomenon in describing its essence [67]. Therefore, in the process of interrogating the phenomenon (i.e., Korean-Canadian immigrants’ experiences with decision-making in primary care), the interviewer and research lead cannot remain neutral or detached from the phenomenon as an individual with experiences and preferences themselves. On the contrary, descriptive (Husserlian) phenomenology states that the researcher must set aside (‘bracket’) their pre-existing assumptions, biases, and experiences with the phenomenon to access the “pure” experience of the phenomenon [68]. As the current study takes a hermeneutic phenomenological approach which believes that the researcher cannot fully and completely separate their pre-existing assumptions, biases, and experiences with the phenomenon, stating (i.e., not bracketing) and reflecting (i.e., engaging in reflexivity) on one’s positionality (including assumptions, biases, and experiences) helps ensure transparency and rigor in this sense.

KGL, the first author and research lead of the study, is a Korean-Canadian economic immigrant, a background that closely aligns with the study participants. This shared identity may influence the research process, shaping the types of interview questions asked, the insights and reflections generated, and the interpretation of data. It may also foster rapport, as participants might feel that KGL can better understand their lived experience. However, KGL’s circumstances may differ from many participants. Having acculturated and attained fluency in English at a young age, she faces minimal linguistic or cultural barriers in healthcare. Her status as a PhD student in medical science may lead providers to take her concerns more seriously, and her education likely contributes to higher health literacy than the general population. Furthermore, possibly due to her background in medical sciences, KGL prefers SDM in her own encounters with physicians. Finally, KGL is a young, relatively healthy individual, which means she has not had to make numerous high-stakes healthcare decisions or those with long-term consequences. This relative lack of experience may predispose her to a bias of naivete, affecting her perspective on the complexities of certain healthcare decisions that participants may face.

To further ensure rigorous reporting, the Consolidated Criteria for Reporting Qualitative Research (COREQ) [69] will be used for this study. While other standard reporting guidelines, such as the Standards for Reporting Qualitative Research (SRQR) [70], are available, the COREQ is specifically designed for qualitative research approaches that employ in-depth and semi-structured interviews [69]. Additionally, given the importance of reflexivity in hermeneutic phenomenology, COREQ’s emphasis on reflexivity makes it particularly suitable for reporting the current study.

Hermeneutic phenomenology aims to uncover the meanings attributed to lived experiences without generating theory. Unlike other qualitative approaches, such as grounded theory, which seek to generate a theory to describe mechanisms of phenomena [71], hermeneutic phenomenology focuses on describing lived experiences and the meanings attributed to them, rather than developing theory, conceptual frameworks, or mechanisms. Therefore, this study does not aim to generate theory.

### Conceptual frameworks

Hermeneutic phenomenology aims to first understand the participant’s worldview and narratives of their experiences, then to interpret it based on the researcher’s understanding. To minimize the influence of the researcher’s prior experiences, assumptions, and theoretical knowledge, analysis will follow a bottom-up (emanating from the data), or inductive, approach to data analysis [72,73]. To achieve this, in addition to employing bracketing and maintaining a reflective diary, this study will employ a conceptual framework relevant to the phenomena of interest (SDM and the therapeutic relationship). These frameworks will not be explicitly referenced during the early- to mid-stages of data analysis, allowing the research team to initially suspend these analytical frameworks to critically assess participants’ experiences [73] to take an inductive approach closer to the data. Later during data analysis, these frameworks will be reintroduced by comparing themes that emerge independently of the framework to those aligned with it. This is important for two reasons: (1) participants’ experiences may not fit into a conceptual framework; and (2) this will enable the researchers to suspend some of their preconceived understandings of the literature and assumptions in initially interpreting the data. This process will enable contrasts and comparisons across themes, facilitating a deeper understanding of the data [73].

The four models of the physician-patient relationship [74] and Elwyn and colleagues’ (2012, 2017) [75,76] three-talk model of SDM (described below) will be used as conceptual frameworks for the therapeutic relationship and SDM, respectively. These conceptual frameworks will be used to represent (i.e., label relevant ideas and themes in the study for communication or writing of the final manuscript) and inform the study (i.e., used in guiding data collection such as in the participant eligibility criteria and interview guide, and interpretation) [73]. Specifically, the four models of the physician-patient relationship and three-talk model of SDM will be used to inform interpretation of emerging themes relevant for the second objective of the proposed study (to investigate Korean immigrant patients’ experiences and preferences regarding decision-making with their primary care providers).

Briefly, the four models of the physician-patient relationship [74] describe paternalistic, informative, interpretive, and deliberative models that differ in (1) the goals of the interaction; (2) the provider’s obligations; (3) the role of the patient’s values; and (4) the understanding of patient autonomy. This framework was chosen to represent the therapeutic relationship because of its compatibility with “sharing” and “caring”, as well as its compatibility with models of decision-making based on the role of the patient’s values and understanding of the patient’s autonomy.

The Three-Talk Model of SDM [75,76] provides a practical framework for SDM and encompasses three steps: (1) Choice talk (introducing choice), (2) Option talk (describing options), and (3) Decision talk (making a preference-concordant decision).

While other models of SDM exist, such as the interprofessional model of SDM [77] and a model by Charles and colleagues (1999) [78], the Three-Talk Model was chosen for this study due to its simplicity and effectiveness in describing the SDM process. This model recognizes that patients may deliberate with friends and family, and that different patients may have varying preferences for their level of involvement in the SDM process, including the option to defer to provider-driven decision-making. This flexibility allows for a nuanced understanding of patient preferences and experiences. Further, given the collectivist nature of Korean immigrants, who may prefer to involve family and friends in their decisions and/or to defer decision-making to their providers, the Three-Talk Model is particularly appropriate for this study.

### Participant inclusion criteria

In order to be eligible to participate in this study, an individual must meet all of the following criteria:

1. Be able to provide informed consent
2. Be aged 18 or older
3. Be able to verbally communicate in English or Korean
4. Be an immigrant who has entered through the economic category and currently have universal healthcare coverage in the province this study is conducted
5. Have immigrated to Canada from South Korea
6. Be ethnically and culturally South Korean
7. Have a family doctor *or* see the same primary care physician at a walk-in *and* have seen the physician at least three [79] times

Immigrants entering Canada through the family or refugee categories experience different migration pressures and policy-related expectations than economic immigrants, which can shape their healthcare experiences [25]. For example, family class immigrants often enter Canada with the endorsement of a sponsor, who is a family member of the individual who is financially responsible for the migrant to ensure they do not require assistance from the government [80]. This can cause pressure for the sponsor and the sponsored individual. Studies report that family-class sponsored immigrants often feel pressured to work and earn money, and may avoid seeking healthcare to avoid burdening their sponsors or family members [25,27,81,82]. On the other hand, many individuals who have refugee status or are refugee claimants do not qualify for universal healthcare coverage in the province where this research will be conducted and therefore are likely to experience reduced healthcare access. Furthermore, refugees migrate to Canada due to circumstances that “push” individuals to emigrate from their homes, such as poverty, violence, political instability, or persecution. Such experiences may influence their level of trust with the Canadian healthcare system and/or the Canadian system as a whole, thereby influencing their healthcare experience and relationships with their providers. Thus, the values and preferences of immigrants from different classes of immigration are likely to differ.

In order to accurately represent immigrants’ experiences, immigrant research should be purposive [25]. Therefore, the current study will explore a single category of immigrants. Although each category of immigrants merits research, this study has specifically chosen the economic category because the majority of immigrants to Canada are from this category [47]. Individuals without permanent residency status will also be excluded, as those without permanent residency do not have universal healthcare coverage in the province this study is conducted and therefore are likely to have unique experiences related to their status. Lastly, to be eligible, individuals must have seen their primary care physicians a minimum of three times. This criterion aligns with widely used measures of the therapeutic relationship, which require at least three sessions to be applicable [79].

### Participant exclusion criteria

An individual who meets any of the following criteria will be excluded from participation in this study:

1. Non-permanent residents (i.e., those who entered Canada through work, study, or temporary resident permits, or who have claimed refugee status) [83]
2. Current or previous immigrant status individuals [83] who have obtained immigrant status through an application class other than the economic application class (i.e., family class, refugee class, or other) [84]
3. Individuals with a personal relationship with the study team.

In this study, a South Korean individual is defined as someone who is both ethnically and culturally South Korean. Therefore, individuals who are ethnically South Korean but culturally associated with another country, such as members of the Korean diaspora that span generations (e.g., Korean-Chinese, also known as Chaoxianzu), or North Koreans (who have distinct cultural and linguistic differences from South Koreans) will be excluded.

### Sampling

The sample of participants recruited will be a mixed heterogeneous sample of sociodemographic profile (e.g., age, education level, gender, English proficiency, and years since landing). Participant recruitment will attempt to represent different genders to account for the influence of gender on the perception of the therapeutic relationship, as well as cultural roles in the social determinants of and experiences of health, which may further influence the therapeutic relationship [27].

Years since landing (in Canada) or English proficiency will not be a criterion but will be collected as demographic data to consider the role of acculturation and language barriers on patient experiences and responses.

Providers (physicians) will not be interviewed because the research question does not attempt to investigate their perception of patient experiences, but rather, patient experiences and perceptions directly.

### Sample size

As phenomenology is a qualitative approach that attempts to identify the essence of human experiences, it involves deep and extensive engagement with participants to explore their lived experiences. Thus, phenomenological studies will have a smaller sample size than other qualitative approaches, such as in grounded theory studies. Creswell and Poth (2018) [64] suggest 10–12 participants for phenomenological studies. Data collection ceases when saturation, or the lack of emergence of any new themes or contributions to the understanding of the study topic, occurs [85]. Therefore, this study will recruit approximately 12 individuals that meet the criteria described. A sample size of 12 individuals is appropriate to achieve the hermeneutic phenomenological research aim of exploring the lived experience of individuals with SDM and the therapeutic relationship, while also large enough to generate a broader scope of data.

### Sampling technique

Hermeneutic phenomenology contends that the meanings attributed to phenomena and experiences are informed by the meaning-maker’s individual historic, cultural, and social background [65,86]. Accordingly, hermeneutic phenomenology often uses a purposive sampling approach to (1) ensure that all participants have experience with the phenomenon; and (2) recruit a diverse sample of individuals to enable collection of rich and unique stories that are informed by their different worldviews [87,88]. Therefore, participants will be recruited using a purposive inclusive criterion sampling approach in combination with a convenience sampling approach.

Inclusive criterion sampling, which is a purposive sampling method, is an approach used to select participants based on specific predetermined criteria or characteristics [86]. This sampling approach will enable the research team to intentionally generate a varied and diverse group of participants that all have experiences of the same phenomenon. For example, as described above, patients under the age of 18 will not be eligible because the therapeutic relationship is likely to be triadic (including physician, patient, and family), resulting in different relational dynamics between physicians and patients. The research team will aim to recruit a sample of participants that are evenly distributed in gender; half newcomer (have been in Canada for less than 10 years [89]), half settled immigrants (have been in Canada for 10 years or more); and half with limited English proficiency. This is in line with a constructivist paradigm, as the researchers will capture variations of experiences with the phenomenon by recruiting participants who vary in characteristics and in their individual experiences. Limited English proficiency indicates those that experience difficulty communicating effectively in English. Therefore, individuals who cannot conduct their interviews in English will be categorized as limited English proficient [90]. During screening, potential participants will be asked about their preferred language for conducting the interview, provided they meet the eligibility criteria.

On the other hand, convenience sampling is an approach wherein individuals who are more accessible to the researcher are more likely to be included [91]. It is a sampling approach that is appropriate for a population that is difficult to reach and retain in research due to linguistic barriers, competing responsibilities, and potential distrust and skepticism about the research process [92,93]. A convenience sampling approach through snowballing is appropriate for this population, where potential research participants can learn of the study through word-of-mouth and reference from trusted community members.

### Active recruitment site

Participants will be actively recruited through a Korean-Canadian Christian church located in a city north of Toronto, within the Greater Toronto Area (GTA). This city is a suburban community with a population of approximately 320,000, and according to the 2021 census, has approximately 4,000 individuals speaking Korean as their mother tongue [94]. The active recruitment site is a multi-generational church that has a Korean-speaking and English-speaking ministry. Many Korean immigrants seek community within Christian churches in the form of a social organization [95,96]. Thus, Korean immigrants have also been recruited from churches in other health-related studies [97–99]. To recruit participants, the research team will collaborate with a church leader, a trusted authority figure in the active site community, who will direct potentially eligible individuals to the research team for screening. Although KGL is also a Korean immigrant, being a first-generation immigrant who migrated at a young age means that her experience may differ significantly from those of recent immigrants, as she has undergone acculturation [92]. Therefore, strategies for recruitment and retention will also draw from community based participatory research approaches to promote culturally appropriate participant engagement by collaborating with community members and trusted organizations [92,93].

### Passive recruitment site

In addition to active recruitment, the research team will recruit participants passively through poster advertisements (both English and Korean) at two major local Korean grocery stores. The first grocery store is a large grocery store chain located in the North York region of Toronto with a population of 647,245, and according to the 2021 census, has approximately 21,865 Korean individuals [100]. The second grocery store is another grocery store chain, located in a city north of Toronto, within the GTA. This city is largely suburban and according to the 2021 census has a population of approximately 202,000 with approximately 6,300 Koreans [101]. The research team will post posters around the entrances and exits of the grocery stores’ building with permission.

The study will also be advertised through print in a local Korean newspaper that serves Korean-Canadians in the GTA and online on a large Korean-Canadian Association’s website. This Korean-Canadian Association is the largest organization serving Korean-Canadians in Canada, and directly serves 120,000 Korean-Canadians living in the GTA.

### Informed consent

Once participant eligibility is confirmed, the participant will be sent an informed consent form and interview guide. Korean-language informed consent forms and interview guides will be available if the potential participant had indicated preference for Korean-language documents and interview. Participants will be encouraged to read and review the informed consent form. Then, the research team will schedule an informed consent discussion together. In this discussion, the informed consent form will be reviewed together with the potential participant and any questions will be addressed. If the potential participant is still interested in participating after this discussion, they will be asked to sign and return the informed consent form via email. For participants who do not sign and return the informed consent form via email (i.e., those that do not have a computer, scanner, etc.), the interviewer (KGL) will receive verbal informed consent after the informed consent discussion. Verbal informed consent will follow a script that was approved by the Research Ethics Boards and will be obtained over the telephone, Zoom, or Microsoft Teams and documented on a form by the researcher obtaining verbal informed consent. Once the individual’s informed consent is received, the research team will contact the participant again to schedule an interview. Interviews will be conducted at a time that is convenient for the participant and various modalities, such as a video or phone call, will be offered. In-person interviews will also be offered and will be held in KGL’s office. As the interviewer, KGL is fluent and can read, write, and speak Korean with little hesitancy. Therefore, an interpreter is not needed.

If a participant fails to attend an interview and is unable to be contacted, a voicemail or email will be sent to the participant thanking them for their time and this individual will be removed as a participant.

Participants who complete the interview will be compensated a one-time payment of $30 CAD for opportunity costs, time, and transportation for participation, while not being financially coerced to participate.

### Data collection procedures

The research team developed an interview guide, which emphasizes (1) assisting participants in constructing their experiences with the therapeutic relationship and SDM; (2) assisting participants in describing their experiential contexts about the therapeutic relationship and SDM; and (3) encouraging participants to reflect on their experiences with the therapeutic relationship and SDM. The interview questions are open-ended, semi-structured, and in-depth (**S1 File**).

Open-ended interview questions will enable the research team to obtain thicker data from the participants, which is important as a phenomenological study exploring participants’ lived experiences. On the other hand, semi-structured interview questions will allow for flexibility during the interview to elicit the essence of the participants’ stories and experiences. For example, the interviewer may wish to stop and/or deviate from the interview guide to ask the participant clarifying or follow-up questions, thus bringing the spoken interview and its essence, experienced by the participant, closer together [102]. The interviewer will encourage the participant to speak as freely as possible, posing questions that allow the participant to become immersed in narrating their experience, such as: Who? What happened next? How did you feel? [102], encouraging the participant to reflect on their experiences during the interview.

The in-depth interviews will take approximately 60 minutes and will be conducted by KGL. During the interview, KGL will keep detailed field notes that describe the participants’ body language, tone, and demeanor, as well as reflections. Additionally, KGL will maintain reflexive diaries about feelings and thoughts throughout the study process. For example, KGL may write thoughts or feelings she had as an acculturated Korean-Canadian immigrant interviewer. Along with the field notes, these reflexive diary entries will be used as supplementary context for data analysis and bracketing [103].

### Data management plan

All information about participants will be kept confidential. Sociodemographic information and personal experiences will be collected as data from participants. No information concerning the study or data collected from participants will be released to any unauthorized third party without prior written approval and notification to the participant.

A password-locked document will be created to store identifying information and stored on a secure server at Mount Sinai Hospital. Identifying information in this document will include the participant’s name, unique ID (e.g., P01 for participant 01), contact information, record of informed consent receipt, preferred language of interview, interview scheduled date, and preference for honoraria. A second document with the participant’s unique ID and responses to eligibility criteria will be maintained. Lastly, a third document will be used to record participant sociodemographic information (e.g., age, marital status, gender, education, etc.) with their unique participant ID. At the beginning of semi-structured interviews, KGL (the interviewer) will review confidentiality and privacy measures with the participant during the informed consent procedure.

Interviews that are conducted virtually (i.e., through a video or audio call) will be audio-recorded (i.e., no video recording) on the platform on which the interview is conducted (e.g., Zoom, Microsoft Teams) and immediately stored on a secure server at Mount Sinai Hospital. A laboratory-owned audio recording device may also be used to record virtual interviews instead of the Zoom or Microsoft Teams recording, if there are any technical issues. Interviews that are conducted in-person will be recorded on a laboratory-owned audio recording device. The audio file from this recording device will then be transferred immediately to a desktop computer at Mount Sinai Hospital, onto a secure server at Mount Sinai Hospital, then permanently deleted from the audio recorder.

KGL will transcribe the audio recordings of interviews, as she is fluent in speaking, writing, and reading Korean and English. Both Korean and English spoken words will be transcribed. All identifying information will be removed from interview transcripts and replaced with pseudonyms or participant unique IDs. From transcription onwards, any and all identifiable information will not be used or included in data analysis, publications, written reports, or products of the study. KGL will then translate Korean transcripts into English. To validate the translations, a subset of audio files (i.e., audio clips that do not include any identifying information) will be sent by secure file transfer to a third-party translation service that will honor data confidentiality as outlined in a service agreement. This third-party service will translate the audio files from Korean to English, which will be compared to KGL’s translations for validation. Demographic characteristics such as age, gender or sex, ethnicity, country of birth, years since landing, English proficiency, and migration status will be included during analysis and in final publications, as they are information that can contextualize participant experiences and study findings. Findings will be shared in aggregated form as themes with de-identified quotes and participant unique IDs. Upon confirmation of accurate transcription, all audio files will be destroyed. Upon completion of data analysis, all identifying data and information will be destroyed. The remaining de-identified data will be stored and maintained for 10 years post-publication, per Sinai Health System (Mount Sinai Hospital) institutional requirements.

### Data analysis procedures

Data collection and analysis will occur simultaneously. As the in-depth interviews will be open-ended and semi-structured, early themes may be identifiable from earlier interviews. Therefore, simultaneous data collection and analysis may allow the interviewer to probe later participants for even richer and deeper data from information gleaned from earlier interviews. Further, thematic analysis will be iterative; codes, individual quotes, passages, and the entire transcript will be reviewed iteratively to deepen understanding, with the researcher’s interpretation continuously informing and being refined by the data.

An inductive analysis approach will be taken to analyze the interview transcripts. During analysis, important quotes about the phenomenon will be identified to generate themes. More specifically, analysis will begin by coding each textual transcript line-by-line (initial coding). During initial coding, statements of significance (i.e., statements that may convey an early emerging theme) will be identified and the list of codes (early themes) will be kept as short as possible [64]. Line-by-line coding will be used because, in line with hermeneutic phenomenology, this enables themes to be generated directly from the data rather than from the researcher’s preconceptions, biases, and interpretations [104]. To avoid inaccurately presenting data, in vivo codes will also be generated to apply the participant’s special terms, as data may be culturally and linguistically nuanced because English will likely be participants’ second language.

Two individuals on the research team will code a minimum of three interviews for researcher triangulation and data validity. To ensure rigor, a codebook describing each code in detail will be developed, and this will be used by all research team members involved in data analysis. In addition to investigator triangulation, data source triangulation will also be used by incorporating field notes and researcher reflexive diaries in analysis [105]. In hermeneutic phenomenology, the researchers’ prior knowledge, biases, assumptions, and experiences actively inform interpretation. Reflexive diary notes will allow authors to explicitly identify and declare these, enabling transparent and reflexive interpretation of participants’ interview data.

Initial codes will then be developed into focused codes, which will span the entire data set but remain specific. These focused codes will be generated by revising categories of initial codes by either combining, dividing, or refining the code categories. Codes of data that do not fit into a conceptual framework and those that were generated guided by a conceptual framework will continue to be kept separate. Focused codes will differentiate between textual descriptions (“what” was experienced) and structural descriptions (“how” it was experienced).

Focused codes will then generate themes by noting patterns and emerging relationships across the focused codes and clustering concepts and ideas. Emerging themes that do not fit into a conceptual framework (i.e., emerged from the data) will be compared and contrasted to those that fit into a conceptual framework (i.e., codes guided by conceptual frameworks) [64]. This comparison will help assess how the data-driven themes relate to established conceptual frameworks. During this stage, frameworks will be used solely as interpretive tools for understanding themes that have emerged directly from the data.

### Current status of the study

As of Fall of 2025, the study has received Research Ethics Board approval from the associated institutions (University of Toronto, REB#: 00047978) (Sinai Health System, REB#: 1271). No participants have yet been recruited. Recruitment is expected to be complete by Spring of 2026, and data collection is expected to complete by early Summer of 2026. The study is expected to be complete in Fall of 2026.

## Discussion

### Knowledge dissemination

This study will explore the experiences and preferences of therapeutic relationships and SDM among Korean immigrant patients and their primary care providers, as well as the factors influencing their perceptions of these concepts. Specifically, this study will explore what the therapeutic relationship signifies to Korean immigrant patients, their understanding of and preferences for their role in SDM and therapeutic relationships, and their expectations from primary care providers.

The knowledge translation objective of this study is to raise awareness about how Korean immigrant patients conceptualize and experience therapeutic relationships and SDM in primary care settings, thereby stimulating further dialogue on designing care specifically tailored to this demographic. Due to its exploratory nature, this study aims to raise awareness rather than advocate for immediate action or structural change. While the study can inform micro-level adjustments (e.g., within individual therapeutic relationships), broader changes at the meso- or macro-levels (e.g., organizational or policy-level) require additional research due to the complexity involved in implementing guidelines, policies, and care structures for South Korean immigrant patients. Despite its focus on raising awareness, this study may offer considerations or suggested practices for engaging with South Korean immigrant patients in primary care based on its findings.

To achieve the knowledge translation goal of raising awareness, the study findings will be shared through various channels. A full-length scientific manuscript will present the findings thematically [106], with de-identified quotes embedded throughout the manuscript. KGL’s personal experiences with the phenomenon will also be reported in the manuscript, aided by reflexive notes and bracketing [64]. Findings of the study will also be shared at conferences and other academic presentations.

For a broader audience, an infographic will be created in both Korean and English, targeting Korean-Canadian immigrants. The infographic will be accessible through the active recruitment site’s website and disseminated through affiliated organizations such as Mount Sinai Hospital and the University of Toronto.

Knowledge users include providers, patients, active recruitment site leadership and staff, as well as other healthcare institutions and organizations, each of whom may derive unique insights relevant to their respective roles and contexts.

### Limitations of study design

This study design has several limitations. First, it does not explore the experiences and perceptions of primary care physicians who provide care to Korean-Canadian immigrant patients. This omission was intentional, as the study aims to center the perspectives of Korean-Canadian immigrant patients themselves rather than the physicians’ interpretations of their experiences. Prior research has shown that healthcare providers may incorrectly assume that East Asian patients prefer passive decision-making styles due to their limited input. However, this lack of input is often attributable to factors such as language barriers, difficulty articulating preferences, time constraints, or power imbalances in the patient-provider relationship rather than an inherent preference for passivity [42].

Second, this study does not incorporate quantitative data collection. While validated SDM measures such as the OPTION [107], SDM-Q-9 [108], or CollaboRATE [109] scales exist, this study is exploratory and does not aim to quantify whether patients are engaging in their preferred decision-making style. Future research could integrate such quantitative methodologies to assess the extent to which patients’ preferences align with actual decision-making practices in clinical encounters.

Third, this study focuses exclusively on Korean-Canadian immigrants, which limits generalizability. Given that hermeneutic phenomenology emphasizes lived experiences shaped by specific social, cultural, and historical contexts, the findings may not be transferable to Korean immigrants in other countries, such as the United States. Healthcare experiences are further shaped by national healthcare systems; for instance, Canada’s publicly funded healthcare system (Medicare) differs significantly from the privatized system in the U.S., where access to providers is often influenced by socioeconomic status and insurance coverage. These systemic differences may lead to distinct patient-provider dynamics across national contexts.

Finally, as this study adopts a hermeneutic phenomenological approach, it does not seek to generate theory, conceptual frameworks, or mechanisms, as grounded theory might. Instead, its findings are intended to deepen understanding of patients’ lived experiences and can serve as a foundation for future research, theoretical development, and program implementation.

### Amendments, premature termination, or suspension of study

Any changes to the study protocol will be submitted as an amendment to the overseeing Research Ethics Board immediately, and these changes will not be implemented until approval is received.

During informed consent, KGL will explain participant rights in detail to explain that they are free to withdraw from taking part in the study at any point throughout the duration of the study upon request without explanation or consequences. Participants who withdraw or terminate their participation will still receive their $30 CAD compensation, and the data they have shared up until that point will be maintained and used, unless indicated or requested otherwise by the participant. Participants will be asked, as described in the informed consent form, to email the research team with a statement indicating their desire to withdraw or terminate their participation. Upon receipt of this email, the research team will ask the participant whether they would still like to receive the final results of the research study during dissemination stages. The participant’s withdrawal/termination of participation, as well as their preference for receiving the results of the study, will be noted.

An individual’s participation in the study may be terminated by the research team if:

- Any medical condition, experience of psychological and/ or emotional distress or situation occurs such that continued participation in the study would not be in the best interest of the participant.
- The participant meets an exclusion criterion (either newly developed or not previously recognized) that precludes further study participation.

This study may be temporarily suspended or prematurely terminated if there is sufficient reasonable cause. If the study is prematurely terminated or suspended, the PI will promptly inform the Research Ethics Board and will provide the reason(s) for the termination or suspension.

## Supporting information

S1 File Interview guide. List of interview questions.
(DOCX)
