# Supplementary material for: Reaching common ground: Protocol for a qualitative study to explore Korean immigrant patient experiences in shared decision-making with primary care physicians
Source: PLoS One. 2026 Aug 7;21(8):e0354905. doi: 10.1371/journal.pone.0354905 (PMC13450765; doi:10.1371/journal.pone.0354905)
Supplement: Supplementary file 1 [file pone.0354905.s001.docx]

**Interview Guide**

***Note:*** *Additional probing questions are italicized.*

**Introduction**

- Welcome, thanks, this interview will take about an hour
- Acknowledge that they already have seen the consent form
- Reminder that this interview is being recorded, this interview will be used for a research project
- At any point if you don’t want to answer any questions, or are not clear what I’m asking, please let me know
- This project is trying to understand South Korean immigrant experiences when they are talking with family doctors, and interactions with family doctors
- We will probably talk a lot about your interactions with your family doctors, maybe a little bit about your health, and your experiences in Canada
- How would you like to be addressed?

**Section A: Demographic Questions**

As part of my research study, I will be asking some questions about you, such as your age, when you came to Canada from South Korea, and so on, to get some context about you. I ask these questions because I want to know how, and if, people with different immigration backgrounds have different experiences with their primary care doctors in Canada. The information you give me about yourself will be connected to the interview responses that you give me today, but nothing that you share with me today will be connected to your name.

You do not need to answer any questions that you don’t wish to. For each question that I ask, you are free to choose “I do not want to answer”, or you can also just say “skip”. Please feel free to ask me any questions or clarify at any point if you are not sure what I am asking.

1. How old are you?

 18-25	66-75

 26-35	76-85

 36-45	85+

 46-55	Prefer not to answer

 56-65

2. What is your marital status?

 Single	Common-law	Separated

 Married	Divorced	Prefer not to answer

3. How would you describe your gender?

 Female	Male	Transwoman
 Transman	Non-binary	Intersex
 Gender neutral	Do not know	Prefer not to answer
 Prefer to self describe: _______________

4. What is the highest level of education you’ve completed? Was that in South Korea, Canada, or elsewhere? *If elsewhere, where?*

 Elementary or Middle School	High-school/secondary school

 College/Diploma/Certificate	Undergraduate degree

 Master’s degree	Professional degree (PhD, JD, MD, etc.)

 Other: __________	Prefer not to answer

5. Which best describes your current employment situation in Canada?

 Unemployed	Self-employed	Full time (40h/week)
 Part time (Less than 40 hours a week)	Retired
 Student	Short-term contract, casual, or seasonal
 Volunteer	Other _____________
 Prefer not to answer

6. What language are you most comfortable communicating in with your primary care doctor?

 Korean	English	Other _______________
 Prefer not to answer

7. How long have you been with your family doctor? What is your doctor’s name? (I ask you for your doctor’s name just for my convenience today when we talk about your experiences)

 Less than 6 months
 6 months to 1 year
 1-3 years
 3-5 years
 >5 years
 Other _____________
 Doctor name (for interview convenience) _____________

8. Do you have any chronic conditions or medication that you take on a regular basis? I am asking this question to understand what you and your doctor might talk about when you meet. You do not have to describe the nature of the condition or the specifics of the medication you take, unless you want to.

 Yes. Additional notes if participant discloses details: __________________
 No	Prefer not to answer

**Section B: Introduction**

1. I want to know about your immigration experience as well. When did you come to Canada, and how have your experiences here been so far? Did you come directly from South Korea?

- *Did you live anywhere else before Ontario, Canada?*
- *Did you have any other family or friends here?*

**Section C: Therapeutic Relationship**

2. Can you tell me about your family doctor/the doctor you see at the walk-in? What are they like, in terms of their personality? How did you find them?
   - Is your provider also Korean? Are they an immigrant?
   - What language do you usually speak with your doctor? How has that experience been like?
3. How would you describe your relationship with your doctor?
   - *Rephrase or probe: If you had to describe your family doctor as playing some kind of role in your life, what would that be? For example, it could be something like a teacher, supporter, mentor, friend, guide, coach, partner, expert, consultant, technician, gatekeeper, mediator*
   - *Could you please explain why you would describe your doctor as playing that kind of role in your life?*
4. Say you had to choose a new family doctor. What’s important for you when choosing a new family doctor?
   - *Rephrase or probe: If there were multiple family doctors in your area, all at similar distance to your house, what other things would you consider in choosing a family doctor? Why?*
5. What has your experiences with family doctors or primary care been like since you have been in Canada?
   - *Do you experience any barriers or challenges in receiving primary healthcare when you need it?*
   - *Do you ever travel back to South Korea to receive healthcare? Why?*
6. Tell me about a time that you saw a [Korean if current is Canadian; Canadian if current is Korean] doctor, either in Korea or Canada. Did it feel different? If so, how, and why?

**Section D: Shared Decision-Making**

I will now be asking you some questions about a time that you had to make a decision about your health with your doctor. I am asking you these questions because I want to understand how you make decisions with your doctor, what your experiences have been, and what your preferences may be when making healthcare decisions.

7. Can you tell me about a time that you had to make a decision that was important to you about our health with your doctor? What was that decision about?
   - *Provide examples if necessary, selecting those that may be most applicable to the participant’s demographic: Some common examples of decisions that are made in primary care can be about*
     i. *vaccinations (flu shot, HPV vaccine, COVID vaccine)*
     ii. *screening tests (mammograms, colonoscopies, prostate health)*
     iii. *lifestyle changes (diet, exercise, smoking cessation)*
     iv. *medication adjustment for conditions like diabetes, hypertension, or asthma*
     v. *treatment options for infections (antibiotics for bacterial infections)*
     vi. *mental health decisions (choosing treatments, such as medications or therapy)*
     vii. *referral decisions (choosing appropriate specialists or the need for a referral to a specialist)*
     viii. *contraceptive choices and menopausal management options*
     ix. *pregnancy-related care (such as prenatal screenings or delivery plans)*
8. How was the need for that decision brought up?
   - How did your doctor explain the different options available to you for the decision?
9. How did you talk about your concerns or your preferences with your doctor?
10. How did the process of deciding go? How was the decision made?
    - Who else besides you and your doctor, if any, were involved in making this decision? Did you talk to any family or friends, search things on the internet, or use any other resources?
    - *Did the doctor give you a recommendation or any advice? Did they ask you what your preference was? How did you feel about this?*
11. What kinds of things are important to you when you make a decision about your health?
    - When you made that decision in the example you gave me, what was most influential for you in coming to that decision?
    - What was your part and your doctor’s part in making that decision?
12. How did you feel about the way the decision was made, and how it happened? If you had to do it again, would you want to do it exactly the same way, or would you want to change it?
13. How do you wish to feel after you make a decision with your doctor? Why is it important for you to feel that way?
14. Going into that interaction, did you have any expectations of your doctor? What were they?

**Section E: Thank and Conclude**

15. Is there anything else that you wish to share with me?
