# Supplementary material for: Reaching common ground: Protocol for a qualitative study to explore Korean immigrant patient experiences in shared decision-making with primary care physicians
Source: PLoS One. 2026 Aug 7;21(8):e0354905. doi: 10.1371/journal.pone.0354905 (PMC13450765; doi:10.1371/journal.pone.0354905)
Supplement: Peer review history

# Peer review: Decision Letter 0

**DOI:** 10.1371/journal.pone.0354905.r001

**Authors:** Ai Theng Cheong

27 Apr 2026

PONE-D-25-59515

Reaching common ground: Protocol for a qualitative study to explore Korean immigrant patient experiences in shared decision-making with primary care physicians

PLOS One

Dear Dr. Maunder,

Thank you for submitting your manuscript to PLOS ONE. After careful consideration, we feel that it has merit but does not fully meet PLOS ONE’s publication criteria as it currently stands. Therefore, we invite you to submit a revised version of the manuscript that addresses the points raised during the review process.

Please submit your revised manuscript by Jun 11 2026 11:59PM. If you will need more time than this to complete your revisions, please reply to this message or contact the journal office at plosone@plos.org. When you're ready to submit your revision, log on to https://www.editorialmanager.com/pone/ and select the 'Submissions Needing Revision' folder to locate your manuscript file.

Please include the following items when submitting your revised manuscript:

- A letter that responds to each point raised by the academic editor and reviewer(s). You should upload this letter as a separate file labeled 'Response to Reviewers'.
- A marked-up copy of your manuscript that highlights changes made to the original version. You should upload this as a separate file labeled 'Revised Manuscript with Track Changes'.
- An unmarked version of your revised paper without tracked changes. You should upload this as a separate file labeled 'Manuscript'.

If you would like to make changes to your financial disclosure, please include your updated statement in your cover letter. Guidelines for resubmitting your figure files are available below the reviewer comments at the end of this letter.

If applicable, we recommend that you deposit your laboratory protocols in protocols.io to enhance the reproducibility of your results. Protocols.io assigns your protocol its own identifier (DOI) so that it can be cited independently in the future. For instructions see: https://journals.plos.org/plosone/s/submission-guidelines#loc-laboratory-protocols. Additionally, PLOS ONE offers an option for publishing peer-reviewed Lab Protocol articles, which describe protocols hosted on protocols.io. Read more information on sharing protocols at https://plos.org/protocols?utm_medium=editorial-email&utm_source=authorletters&utm_campaign=protocols.

As the corresponding author, your ORCID iD is verified in the submission system and will appear in the published article. PLOS supports the use of ORCID, and we encourage all coauthors to register for an ORCID iD and use it as well. Please encourage your coauthors to verify their ORCID iD within the submission system before final acceptance, as unverified ORCID iDs will not appear in the published article. *Only*  the individual author can complete the verification step; PLOS staff *cannot*  verify ORCID iDs on behalf of authors.

We look forward to receiving your revised manuscript.

Kind regards,

Ai Theng Cheong

Academic Editor

PLOS One

Journal Requirements:

When submitting your revision, we need you to address these additional requirements.

1. Please ensure that your manuscript meets PLOS ONE's style requirements, including those for file naming. The PLOS ONE style templates can be found at

https://journals.plos.org/plosone/s/file?id=wjVg/PLOSOne_formatting_sample_main_body.pdf and https://journals.plos.org/plosone/s/file?id=ba62/PLOSOne_formatting_sample_title_authors_affiliations.pdf

2. PLOS requires an ORCID iD for the corresponding author in Editorial Manager on papers submitted after December 6th, 2016. Please ensure that you have an ORCID iD and that it is validated in Editorial Manager. To do this, go to ‘Update my Information’ (in the upper left-hand corner of the main menu), and click on the Fetch/Validate link next to the ORCID field. This will take you to the ORCID site and allow you to create a new iD or authenticate a pre-existing iD in Editorial Manager.

3. Your ethics statement should only appear in the Methods section of your manuscript. If your ethics statement is written in any section besides the Methods, please delete it from any other section.

4. Please provide a complete Data Availability Statement in the submission form, ensuring you include all necessary access information or a reason for why you are unable to make your data freely accessible. If your research concerns only data provided within your submission, please write "All data are in the manuscript and/or supporting information files" as your Data Availability Statement.

5. We note that the grant information you provided in the ‘Funding Information’ and ‘Financial Disclosure’ sections do not match.

When you resubmit, please ensure that you provide the correct grant numbers for the awards you received for your study in the ‘Funding Information’ section.

6. Please amend your authorship list in your manuscript file to include author Robert Maunder.

7. Please amend the manuscript submission data (via Edit Submission) to include author Robert G. Maunder.

8. Please include captions for your Supporting Information files at the end of your manuscript, and update any in-text citations to match accordingly. Please see our Supporting Information guidelines for more information: http://journals.plos.org/plosone/s/supporting-information.

9. If the reviewer comments include a recommendation to cite specific previously published works, please review and evaluate these publications to determine whether they are relevant and should be cited. There is no requirement to cite these works unless the editor has indicated otherwise.

[Note: HTML markup is below. Please do not edit.]

Reviewers' comments:

Reviewer's Responses to Questions

**Comments to the Author**

1. Does the manuscript provide a valid rationale for the proposed study, with clearly identified and justified research questions?

The research question outlined is expected to address a valid academic problem or topic and contribute to the base of knowledge in the field.

Reviewer #1: Yes

Reviewer #2: Yes

**********

2. Is the protocol technically sound and planned in a manner that will lead to a meaningful outcome and allow testing the stated hypotheses?

The manuscript should describe the methods in sufficient detail to prevent undisclosed flexibility in the experimental procedure or analysis pipeline, including sufficient outcome-neutral conditions (e.g. necessary controls, absence of floor or ceiling effects) to test the proposed hypotheses and a statistical power analysis where applicable. As there may be aspects of the methodology and analysis which can only be refined once the work is undertaken, authors should outline potential assumptions and explicitly describe what aspects of the proposed analyses, if any, are exploratory.

Reviewer #1: Yes

Reviewer #2: Yes

**********

3. Is the methodology feasible and described in sufficient detail to allow the work to be replicable?

Descriptions of methods and materials in the protocol should be reported in sufficient detail for another researcher to reproduce all experiments and analyses. The protocol should describe the appropriate controls, sample size calculations, and replication needed to ensure that the data are robust and reproducible.

Reviewer #1: Yes

Reviewer #2: Yes

**********

4. Have the authors described where all data underlying the findings will be made available when the study is complete?

The PLOS Data policy requires authors to make all data underlying the findings described in their manuscript fully available without restriction, with rare exception, at the time of publication. The data should be provided as part of the manuscript or its supporting information, or deposited to a public repository. For example, in addition to summary statistics, the data points behind means, medians and variance measures should be available. If there are restrictions on publicly sharing data—e.g. participant privacy or use of data from a third party—those must be specified.

Reviewer #1: Yes

Reviewer #2: Yes

**********

5. Is the manuscript presented in an intelligible fashion and written in standard English?

PLOS ONE does not copyedit accepted manuscripts, so the language in submitted articles must be clear, correct, and unambiguous. Any typographical or grammatical errors should be corrected at revision, so please note any specific errors here.

Reviewer #1: Yes

Reviewer #2: Yes

**********

6. Review Comments to the Author

Please use the space provided to explain your answers to the questions above and, if applicable, provide comments about issues authors must address before this protocol can be accepted for publication. You may also include additional comments for the author, including concerns about research or publication ethics.

You may also provide optional suggestions and comments to authors that they might find helpful in planning their study.

(Please upload your review as an attachment if it exceeds 20,000 characters)

Reviewer #1: The protocol is clear and well -written. The theoretical approaches are described well and adequate reflexivity on the researchers own identity as a Korean background immigrant is described.

It is hard to pin down what might emerge from focusing on SDM among a diverse community that cannot be defined clearly in terms of culture (ie the researcher mentions that Korean communities are non-monolithic) in terms of SDM practice or experience in primary care. As such, the phenomena of interest (Korean culture, in an immigrant community and how SDM is experienced in primary care) might be too vague or diluted for any patterns to emerge from the study.

The topic guide could be adjusted to include some more specific elements of Korean immigrant culture. It currently asks about doctor-patient matching, and decision making about health decisions, which might be too obliquely related to cultural or community influences of SDM if these are just described. Maybe consider some prompts about Korean immigrant culture and views of doctors and medicine?

Reviewer #2: Dear Author,

Good effort and interesting study.

There are a few comments that I have highlighted but mainly I feel that the author can be more concise in the introduction, focusing on SDM as the main topic. The description on caring and sharing should be explicit into the context of SDM in therapeutic dr-patient relationship. Some of the good statemebts are embeded within the winding explanation of SDM and immigrants and how this shapes future therapeutic relationship.

The gap needs reference to highlight the sparsity of literatures among these group.

2. Objective 2 requires specific area of decision making

3. I feel a lot of explanation regarding theories underpinning the research are generic and very theoretical. Please relate those with the core matter.

4. Interpretivist framework to me is more of a framework supporting the design of hermeneustic phenomenology rather that the theory to underpin your research phenomena.

6. It would be better that the design is explained explicitly in comparison to descriptive phenomenology rather than grounded theory. I feel the readers would appreciate a more explicit description of the hermeneustic characters like; non bracketing and reflexivity ( you did at the end regarding the first author background) and the speculated hidden meaning like fear, trust from the participant's experience.

7. No topic guide is inserted

8. Please elaborate how in the analysis will the hidden meaning be explored.

**********

7. PLOS authors have the option to publish the peer review history of their article (what does this mean?). If published, this will include your full peer review and any attached files.

If you choose “no”, your identity will remain anonymous but your review may still be made public.

**Do you want your identity to be public for this peer review?**  For information about this choice, including consent withdrawal, please see our Privacy Policy.

Reviewer #1: **Yes:** LEE YEW KONG

Reviewer #2: No

**********

[NOTE: If reviewer comments were submitted as an attachment file, they will be attached to this email and accessible via the submission site. Please log into your account, locate the manuscript record, and check for the action link "View Attachments". If this link does not appear, there are no attachment files.]

To ensure your figures meet our technical requirements, please review our figure guidelines: https://journals.plos.org/plosone/s/figures

You may also use PLOS’s free figure tool, NAAS, to help you prepare publication quality figures: https://journals.plos.org/plosone/s/figures#loc-tools-for-figure-preparation.

NAAS will assess whether your figures meet our technical requirements by comparing each figure against our figure specifications.

# Author comment: Author response to Decision Letter 1

**DOI:** 10.1371/journal.pone.0354905.r002

4 Jun 2026

Dear Dr. Cheong, Dr. Lee, and Reviewer #2,

Reviewer's Responses to Questions

Comments to the Author

1. Does the manuscript provide a valid rationale for the proposed study, with clearly identified and justified research questions?

The research question outlined is expected to address a valid academic problem or topic and contribute to the base of knowledge in the field.

Reviewer #1: Yes

Reviewer #2: Yes

2. Is the protocol technically sound and planned in a manner that will lead to a meaningful outcome and allow testing the stated hypotheses?

The manuscript should describe the methods in sufficient detail to prevent undisclosed flexibility in the experimental procedure or analysis pipeline, including sufficient outcome-neutral conditions (e.g. necessary controls, absence of floor or ceiling effects) to test the proposed hypotheses and a statistical power analysis where applicable. As there may be aspects of the methodology and analysis which can only be refined once the work is undertaken, authors should outline potential assumptions and explicitly describe what aspects of the proposed analyses, if any, are exploratory.

Reviewer #1: Yes

Reviewer #2: Yes

3. Is the methodology feasible and described in sufficient detail to allow the work to be replicable?

Descriptions of methods and materials in the protocol should be reported in sufficient detail for another researcher to reproduce all experiments and analyses. The protocol should describe the appropriate controls, sample size calculations, and replication needed to ensure that the data are robust and reproducible.

Reviewer #1: Yes

Reviewer #2: Yes

4. Have the authors described where all data underlying the findings will be made available when the study is complete?

The PLOS Data policy requires authors to make all data underlying the findings described in their manuscript fully available without restriction, with rare exception, at the time of publication. The data should be provided as part of the manuscript or its supporting information, or deposited to a public repository. For example, in addition to summary statistics, the data points behind means, medians and variance measures should be available. If there are restrictions on publicly sharing data—e.g. participant privacy or use of data from a third party—those must be specified.

Reviewer #1: Yes

Reviewer #2: Yes

5. Is the manuscript presented in an intelligible fashion and written in standard English?

PLOS ONE does not copyedit accepted manuscripts, so the language in submitted articles must be clear, correct, and unambiguous. Any typographical or grammatical errors should be corrected at revision, so please note any specific errors here.

Reviewer #1: Yes

Reviewer #2: Yes

6. Review Comments to the Author

Please use the space provided to explain your answers to the questions above and, if applicable, provide comments about issues authors must address before this protocol can be accepted for publication. You may also include additional comments for the author, including concerns about research or publication ethics.

You may also provide optional suggestions and comments to authors that they might find helpful in planning their study.

(Please upload your review as an attachment if it exceeds 20,000 characters)

Reviewer #1:

The protocol is clear and well -written. The theoretical approaches are described well and adequate reflexivity on the researchers own identity as a Korean background immigrant is described.

It is hard to pin down what might emerge from focusing on SDM among a diverse community that cannot be defined clearly in terms of culture (ie the researcher mentions that Korean communities are non-monolithic) in terms of SDM practice or experience in primary care. As such, the phenomena of interest (Korean culture, in an immigrant community and how SDM is experienced in primary care) might be too vague or diluted for any patterns to emerge from the study.

• Thank you for this thoughtful comment. We acknowledge the concern that patterns may be difficult to discern given the diversity of Korean immigrant experiences with SDM in Canadian primary care. This heterogeneity, however, was not only anticipated but intentionally accounted for in our methodological approach. We purposefully employed a hermeneutic phenomenological framework, which recognizes that everyone’s understanding of the world (and in this case, their experience of SDM) is uniquely shaped by their personal history, cultural background, and lived experience. Therefore, we believe this framework embraces, rather than conflicts, with the diversity in the population. The goal of our study was not to identify a single “Korean-Canadian experience” of SDM, but to develop a rich, nuanced understanding of how SDM is experienced across a range of individual perspectives within the community. We recognize the need to acknowledge this nuance, and therefore, have added a line in the Methods: “However, as hermeneutic phenomenology claims that meaning is found as people construct the world and relevant knowledge based on their own background and experiences [68], even those who share similar cultural, ethnic, and immigration backgrounds may have different experiences and preferences relating to decision-making and the therapeutic relationship.”.

To further ensure coherence within this diversity, we also selected our inclusion and exclusion criteria to recruit participants with comparably similar immigration trajectories. For example, by focusing on economic immigrants, whose decision to immigrate was more likely driven by “pull” factors than “push” factors (as opposed to refugees, who may migrate due to “push” factors), we sought to introduce a meaningful point of commonality in participant backgrounds.

The topic guide could be adjusted to include some more specific elements of Korean immigrant culture. It currently asks about doctor-patient matching, and decision making about health decisions, which might be too obliquely related to cultural or community influences of SDM if these are just described. Maybe consider some prompts about Korean immigrant culture and views of doctors and medicine?

• Thank you for this helpful suggestion. We agree that more explicit prompts about Korean immigrant culture would strengthen the topic guide (interview guide). We want to note that, as a semi-structured interview, we will not adhere strictly to the guide and will probe for cultural context throughout.

We believe several existing questions are already well-positioned to elicit participants’ views on Korean culture, doctors, and medicine. For example, the question “I want to know about your immigration experience as well. When did you first come to Canada, and how have your experiences here been so far?” may probe participants to share their views on Korean immigrant culture. Views and comparisons between Canadian and Korean culture, doctors, and medicine may arise in questions such as “Say you had to choose a new family doctor. What’s important to you when choosing a new family doctor?” (e.g., they may prefer a Korean doctor compared to a non-Korean doctor for x, y, z, reasons), “Tell me about a time that you saw a [Korean if current is Canadian; Canadian if current is Korean] doctor, either in Korea or Canada. Did it feel different? If so, how, and why?”, and an italicized probing question, “Do you ever travel back to South Korea to receive healthcare? Why?” (which may prompt participants to share any views and/or experiences of doctors and medicine in Korea compared to that in Canada).

Reviewer #2:

Dear Author,

Good effort and interesting study.

There are a few comments that I have highlighted but mainly I feel that the author can be more concise in the introduction, focusing on SDM as the main topic. The description on caring and sharing should be explicit into the context of SDM in therapeutic dr-patient relationship. Some of the good statemebts are embeded within the winding explanation of SDM and immigrants and how this shapes future therapeutic relationship.

The gap needs reference to highlight the sparsity of literatures among these group.

• Thank you for this suggestion. We have revised the introduction to be more concise, focusing on SDM as the main topic and making more explicit the concepts of “caring” and “sharing” in describing SDM within a therapeutic relationship.

2. Objective 2 requires specific area of decision making

• Thank you for this comment. Objective 2 is intentionally broad, as we were interested in exploring the entire decision-making process, from identifying the need to make a decision, deliberation and weighing options, to reaching a final decision. We have clarified this in the manuscript. We also did not specify a particular type of decision-making (e.g., paternalistic, informed, or shared) as our goal was to elicit participants’ own experiences and preferences without presupposing a framework.

3. I feel a lot of explanation regarding theories underpinning the research are generic and very theoretical. Please relate those with the core matter.

• Thank you for this comment. We agree, and we have removed descriptions of theory, epistemology, and ontology, while keeping only descriptions that relate directly to decision-making and the current study. For example, we have clarified what the ‘descriptions of meanings’ and ‘phenomenon of interest’ is in this study: “In doing so, the findings of a phenomenological study is a collection of descriptions of meanings (i.e., experiences, preferences) individuals attribute to their lived experiences of a concept or phenomenon [77] (i.e., decision-making in primary care).”

4. Interpretivist framework to me is more of a framework supporting the design of hermeneustic phenomenology rather that the theory to underpin your research phenomena.

• We agree, and we have removed the theoretical descriptions of interpretivist frameworks of inquiry. We have maintained only one mention of interpretivist frameworks, which describes that in studies that employ this framework of inquiry (such as hermeneutic phenomenological studies as this), validity and reliability can be achieved through reporting of positionality and reflexivity: “In qualitative research that takes an interpretivist framework of inquiry, validity and reliability are achieved through the rigor, trustworthiness, credibility, and authenticity of both the research and the researcher [68]. Therefore, reporting and deeply reflecting on the researcher’s experiences and assumptions are critical for the research process. The first author and research lead of the study will report their positionality. Hermeneutic phenomenology asserts that the observer or researcher cannot fully remove themselves from the phenomenon in describing its essence [81]”

6. It would be better that the design is explained explicitly in comparison to descriptive phenomenology rather than grounded theory. I feel the readers would appreciate a more explicit description of the hermeneustic characters like; non bracketing and reflexivity ( you did at the end regarding the first author background) and the speculated hidden meaning like fear, trust from the participant's experience.

• Thank you for this suggestion. Our intention was to highlight that our study does not intend to generate theory, and therefore we used grounded theory as a comparison. However, we understand the point in that readers may appreciate a more explicit description of key hermeneutic characters. Therefore, we have revised the manuscript to describe such components (e.g., non-bracketing, reflexivity): “On the contrary, descriptive (Husserlian) phenomenology states that the researcher must set aside (‘bracket’) their pre-existing assumptions, biases, and experiences with the phenomenon to access the “pure” experience of the phenomenon. As the current study takes a hermeneutic phenomenological approach which believes that the researcher cannot fully and completely separate their pre-existing assumptions, biases, and experiences with the phenomenon, stating (i.e., not bracketing) and reflecting (i.e., engaging in reflexivity) on one’s positionality (including assumptions, biases, and experiences) helps ensure transparency and rigor in this sense.”

7. No topic guide is inserted

• We have included the topic guide (interview guide) as Supporting information (S1 File).

8. Please elaborate how in the analysis will the hidden meaning be explored.

• The hidden meaning (or the process of interpretation) will be explored primarily through thematic analysis, reflexive diary notes, and reflexivity. Thematic analysis will be iterative; individual quotes, passages, and the entire transcript will be reviewed iteratively as codes are combined and revised to inform themes. Further, as the authors’ prior knowledge, biases, assumptions, and experiences actively inform interpretation, a set of reflexive diary notes will be kept wherein the interviewer (KGL) maintains notes on reflections, emotions, thoughts, observations, and interpretations during and immediately after the interview, as well as during the coding and analysis process. This set of reflexive notes will inform reflexive interpretation by enabling the authors to explicitly identify and declare experiences, biases, and assumptions that inform interpretation of participants’ interview data. We have revised the manuscript to reflect this additional detail: “Further, thematic analysis will be iterative; codes, individual quotes, passages, and the entire transcript will be reviewed iteratively to deepen understanding, with the researcher’s interpretation continuously informing and being refined by the data.” and “hermeneutic phenomenology, the researchers’ prior knowledge, biases, assumptions, and experiences actively inform interpretation. Reflexive diary notes will allow authors to explicitly identify and declare these, enabling transparent and reflexive interpretation of participants’ interview data.”

# Peer review: Decision Letter 1

**DOI:** 10.1371/journal.pone.0354905.r003

**Authors:** Ai Theng Cheong

14 Jul 2026

Reaching common ground: Protocol for a qualitative study to explore Korean immigrant patient experiences in shared decision-making with primary care physicians

PONE-D-25-59515R1

Dear Dr. Maunder,

We’re pleased to inform you that your manuscript has been judged scientifically suitable for publication and will be formally accepted for publication once it meets all outstanding technical requirements.

Within one week, you’ll receive an e-mail detailing the required amendments. When these have been addressed, you’ll receive a formal acceptance letter and your manuscript will be scheduled for publication.

An invoice will be generated when your article is formally accepted. Please note, if your institution has a publishing partnership with PLOS and your article meets the relevant criteria, all or part of your publication costs will be covered. Please make sure your user information is up-to-date by logging into Editorial Manager at Editorial Manager® and clicking the ‘Update My Information' link at the top of the page. For questions related to billing, please contact billing support.

If your institution or institutions have a press office, please notify them about your upcoming paper to help maximize its impact. If they’ll be preparing press materials, please inform our press team as soon as possible -- no later than 48 hours after receiving the formal acceptance. Your manuscript will remain under strict press embargo until 2 pm Eastern Time on the date of publication. For more information, please contact onepress@plos.org.

Kind regards,

Ai Theng Cheong

Academic Editor

PLOS One

Additional Editor Comments (optional):

Reviewers' comments:

Reviewer's Responses to Questions

**Comments to the Author**

1. Does the manuscript provide a valid rationale for the proposed study, with clearly identified and justified research questions?

The research question outlined is expected to address a valid academic problem or topic and contribute to the base of knowledge in the field.

Reviewer #1: Yes

**********

2. Is the protocol technically sound and planned in a manner that will lead to a meaningful outcome and allow testing the stated hypotheses?

The manuscript should describe the methods in sufficient detail to prevent undisclosed flexibility in the experimental procedure or analysis pipeline, including sufficient outcome-neutral conditions (e.g. necessary controls, absence of floor or ceiling effects) to test the proposed hypotheses and a statistical power analysis where applicable. As there may be aspects of the methodology and analysis which can only be refined once the work is undertaken, authors should outline potential assumptions and explicitly describe what aspects of the proposed analyses, if any, are exploratory.

Reviewer #1: Yes

**********

3. Is the methodology feasible and described in sufficient detail to allow the work to be replicable?

Descriptions of methods and materials in the protocol should be reported in sufficient detail for another researcher to reproduce all experiments and analyses. The protocol should describe the appropriate controls, sample size calculations, and replication needed to ensure that the data are robust and reproducible.

Reviewer #1: Yes

**********

4. Have the authors described where all data underlying the findings will be made available when the study is complete?

The PLOS Data policy requires authors to make all data underlying the findings described in their manuscript fully available without restriction, with rare exception, at the time of publication. The data should be provided as part of the manuscript or its supporting information, or deposited to a public repository. For example, in addition to summary statistics, the data points behind means, medians and variance measures should be available. If there are restrictions on publicly sharing data—e.g. participant privacy or use of data from a third party—those must be specified.

Reviewer #1: Yes

**********

5. Is the manuscript presented in an intelligible fashion and written in standard English?

PLOS ONE does not copyedit accepted manuscripts, so the language in submitted articles must be clear, correct, and unambiguous. Any typographical or grammatical errors should be corrected at revision, so please note any specific errors here.

Reviewer #1: Yes

**********

6. Review Comments to the Author

Please use the space provided to explain your answers to the questions above and, if applicable, provide comments about issues authors must address before this protocol can be accepted for publication. You may also include additional comments for the author, including concerns about research or publication ethics.

You may also provide optional suggestions and comments to authors that they might find helpful in planning their study.

(Please upload your review as an attachment if it exceeds 20,000 characters)

Reviewer #1: The authors have addressed my reviewer comments adequately. Specifically, some adjustments to more clearly elicit the Korean immigrant aspect of the study have been added to the methods as well as more specific prompts in the topic guide. As the author has mentioned, the topic guide is not a fixed questionnaire, and a diverse set of possible answers is possible with the current approach.

**********

7. PLOS authors have the option to publish the peer review history of their article (what does this mean?). If published, this will include your full peer review and any attached files.

If you choose “no”, your identity will remain anonymous but your review may still be made public.

**Do you want your identity to be public for this peer review?**  For information about this choice, including consent withdrawal, please see our Privacy Policy.

Reviewer #1: **Yes:** LEE YEW KONG

**********

# Editor report: Acceptance letter

**DOI:** 10.1371/journal.pone.0354905.r004

**Authors:** Ai Theng Cheong

PONE-D-25-59515R1

PLOS One

Dear Dr. Maunder,

I'm pleased to inform you that your manuscript has been deemed suitable for publication in PLOS One. Congratulations! Your manuscript is now being handed over to our production team.

At this stage, our production department will prepare your paper for publication. This includes ensuring the following:

* All references, tables, and figures are properly cited

* All relevant supporting information is included in the manuscript submission,

* There are no issues that prevent the paper from being properly typeset

You will receive further instructions from the production team, including instructions on how to review your proof when it is ready. Please keep in mind that we are working through a large volume of accepted articles, so please give us a few days to review your paper and let you know the next and final steps.

Lastly, if your institution or institutions have a press office, please let them know about your upcoming paper now to help maximize its impact. If they'll be preparing press materials, please inform our press team within the next 48 hours. Your manuscript will remain under strict press embargo until 2 pm Eastern Time on the date of publication. For more information, please contact onepress@plos.org.

You will receive an invoice from PLOS for your publication fee after your manuscript has reached the completed accept phase. If you receive an email requesting payment before acceptance or for any other service, this may be a phishing scheme. Learn how to identify phishing emails and protect your accounts at https://explore.plos.org/phishing.

If we can help with anything else, please email us at customercare@plos.org.

Thank you for submitting your work to PLOS ONE and supporting open access.

Kind regards,

PLOS ONE Editorial Office Staff

on behalf of

Dr. Ai Theng Cheong

Academic Editor

PLOS One
